# 肺癌类器官在肺癌精准医学的应用及其研究进展

**DOI:** 10.3779/j.issn.1009-3419.2024.106.07

**Published:** 2024-04-20

**Authors:** Zhicheng HUANG, Bowen LI, Yadong WANG, Jianchao XUE, Zewen WEI, Naixin LIANG, Shanqing LI

**Affiliations:** ^1^100730 北京，中国医学科学院，北京协和医学院，北京协和医院胸外科; ^1^Department of Thoracic Surgery, Peking Union Medical College Hospital, Chinese Academy of Medical Sciences (CAMS) and Peking Union Medical College (PUMC), Beijing 100730, China; ^2^100081 北京，北京理工大学医学技术学院生物医学工程系; ^2^Department of Biomedical Engineering, School of Medical Technology, Beijing Institute of Technology, Beijing 100081, China

**Keywords:** 肺肿瘤, 类器官, 精准医学, Lung neoplasms, Organoid, Precision medicine

## Abstract

分子检测技术的不断进步极大地推动了肺癌精准医学的发展，但是肿瘤的异质性与肿瘤的转移、复发和耐药密切相关，并且具有同一基因突变的不同肺癌患者采用不同的治疗策略会呈现出一定的疗效差异，因此现代精准医学的发展亟需通过个性化的肿瘤模型来精准制定个性化的治疗策略。肺癌类器官（lung cancer organoid, LCO）能够高度模拟肿瘤在体内的生物学特征，促进抗体药物偶联物等创新药物在肺癌精准医学的应用。随着LCO与肿瘤微环境共培养模型和微流控芯片等组织工程技术的发展，LCO可以更好地保留肿瘤组织的生物学特征和功能，进一步提高高通量和自动化药物敏感性实验。本文结合了最新的LCO研究进展，总结了LCO在肺癌精准医学中的应用进展与挑战。

## Abstract (en)

The continuous advancement of molecular detection technology has greatly propelled the development of precision medicine for lung cancer. However, tumor heterogeneity is closely associated with tumor metastasis, recurrence, and drug resistance. Additionally, different lung cancer patients with the same genetic mutation may exhibit varying treatment responses to different therapeutic strategies. Therefore, the development of modern precision medicine urgently requires the precise formulation of personalized treatment strategies through personalized tumor models. Lung cancer organoid (LCO) can highly simulate the biological characteristics of tumor in vivo, facilitating the application of innovative drugs such as antibody-drug conjugate in precision medicine for lung cancer. With the development of co-culture model of LCO with tumor microenvironment and tissue engineering technology such as microfluidic chip, LCO can better preserve the biological characteristics and functions of tumor tissue, further improving high-throughput and automated drug sensitivity experiment. In this review, we combine the latest research progress to summarize the application progress and challenges of LCO in precision medicine for lung cancer.

## 1 肺癌类器官的应用背景

肺癌是世界上最常见的癌症之一，并且是全球恶性肿瘤相关死亡的主要原因。其中非小细胞肺癌（non-small cell lung cancer, NSCLC）是肺癌中最常见的病理类型，约占所有肺癌的85%^[1]^。近年来，分子检测技术的进步极大地推动了基于基因组学的肺癌精准医学的发展^[2]^，但是肿瘤的异质性与肿瘤的复发转移和耐药密切相关，且具有同一基因突变的不同肺癌患者采用不同的治疗策略会呈现出一定的疗效差异^[3]^，因此基于基因组学的精准医学存在其固有的局限性，现代精准医学的发展亟需通过一种能反映原发肿瘤生物学特征的体外模型来精准制定个性化的治疗策略。肺癌类器官（lung cancer organoid, LCO）是指利用患者来源的肿瘤细胞进行体外三维培养而形成的具有一定空间结构的组织类似物，并能分化为具有特定功能的细胞群，可以模拟肺癌在体内的生物学行为，通过重现肺癌发生发展的病理生理特征来弥合基因型和表型之间的差距^[4]^，因此LCO可以作为肺癌精准医学发展的有效体外模型^[5]^。本文结合了本领域的最新研究进展来探讨LCO在肺癌精准治疗中的应用以及最新的研究进展与挑战。

## 2 不同肿瘤模型的优缺点比较

常用的临床前肿瘤模型主要包括：肿瘤细胞系培养模型（cancer cell line, CCL）、肿瘤细胞原代培养模型（primary tumor cell, PTC）、患者肿瘤来源异种移植模型（patient-derived xenograft, PDX）、细胞系来源异种移植瘤模型（cell line-derived xenograft, CDX）、基因工程小鼠模型（genetically engineered mouse model, GEMM）和LCO模型。传统的肿瘤模型在研究疾病的发生发展机制中发挥了重要的作用，但是由于传统的肿瘤模型不能准确保留原发肿瘤的遗传特征，缺乏人体特有的免疫微环境，无法准确模拟肿瘤细胞与人体基质细胞之间的相互作用，从而限制了相关临床转化研究（表1）^[6⇓⇓⇓-10]^。相较之下，用于构建LCO的标本来源丰富，构建周期短，成功率高，具有高度遗传保真度，能够高度模拟患者的组织细胞结构和生物学特征，在肺癌新药研发与测试中起到了非常重要的作用^[11,12]^。

**表 1:** 不同肿瘤模型的优缺点比较

Model type	Advantage	Limitation
Cancer cell line^[6]^	▪ Comprising pure population of tumor cells ▪ Can be selected based on phenotype or genotype	▪ Not possessing the human immune microenvironment ▪ Incompletely preserving the characteristics of the primary tumor
Primary tumor cell^[7]^	▪ Maintaining the original genetic characteristics of tumor cells ▪ Having abundant specimen sources	▪ Not possessing the human immune microenvironment ▪ Lacking intercellular interactions
Patient-derived xenograft^[8]^	▪ Preserving the genetic characteristics of the primary tumor	▪ Time-consuming and costly ▪ Exhibiting non-species-specific interaction and genetic drift between tumor and host
Cell line-derived xenograft^[9]^	▪ Enabling assessment of tumor development and progression	▪ Not possessing the human immune microenvironment ▪ Incompletely preserving the characteristics of the primary tumor
Genetically engineered mouse model^[10]^	▪ Belonging to in situ tumor ▪ Possessing a complete immune system	▪ Time-consuming and costly ▪ Not possessing the human immune microenvironment

## 3 LCO的构建方法

### 3.1 肺癌类器官的构建与验证

LCO的构建暂时还没有统一的标准程序，培养基的组成成分对LCO的成功构建至关重要，目前通常使用含有Matrigel的基质胶。Matrigel是来源于EHS（Engelbreth-Holm-Swarm）小鼠肉瘤的基底膜基质提取物（basement membrane extract, BME），主要由细胞外基质（extracellular matrix, ECM）成分组成，包含有胶原蛋白、层黏连蛋白、蛋白聚糖与多种生长因子等。ECM对细胞的黏附、功能和组织稳定至关重要，可以通过激活信号通路，直接或间接调节干细胞增殖分化^[13⇓-15]^。Endo等^[16]^构建了第一个LCO的三维培养系统，他们将新鲜的肺癌手术标本切碎消化处理后在含有牛血清白蛋白（bovine serum albumin, BSA）、Neuregulin-1和Matrigel的DMEM/F12培养基中进行培养，该研究团队的LCO构建策略达到了80%的成功率。LCO的成功构建往往要求在取样时有足够多的活性肿瘤细胞，但在某些情况下无法获得足够多的肺癌组织样本，因此许多研究者尝试使用支气管肺泡灌洗液或循环肿瘤细胞（circulating tumor cell, CTC）进行培养，但由于CTC在血液中的含量较少导致LCO培养的成功率相对较低，如何从患者血液样本中迅速富集和分离活性的CTC成为目前的挑战之一^[17⇓-19]^。培养的LCO是否能够准确再现原发肿瘤的组织学和基因学特征对于后续的研究也非常重要，因此LCO构建之后有必要进行相关的验证工作，目前常用的验证方法包括基于形态学的苏木素-伊红（hematoxylin and eosin, HE）染色和免疫组化来区分正常类器官和肿瘤类器官^[20]^。然而，在培养过程中不同的LCO在显微镜下可能呈现出不同的组织形态，因此单纯通过显微镜下的LCO形态学结构很难对构建的类器官作出准确的验证与评估。为了解决这一问题，第二代测序（next-generation sequencing, NGS）和单细胞RNA测序（single-cell RNA sequencing, scRNA-seq）能够在肺部肿瘤的基因组和转录组层面验证LCO的生物学特征，助力肺癌精准医学的发展^[21,22]^。

### 3.2 LCO与肿瘤微环境（tumor microenvironment, TME）的共培养模型的构建

TME包含有ECM和基质细胞，肿瘤细胞与TME的相互作用与肿瘤的增殖、耐药、免疫逃逸和转移扩散等有着密切的联系^[23]^。传统的临床前肿瘤模型不能准确地反映原发肿瘤的实际表型并对其进行干预调控，通过在LCO培养基中添加基质细胞（包括内皮细胞、免疫细胞和成纤维细胞）来实现与TME共培养模型的构建，可以有效实现对细胞间的相互作用进行体外观察和定量研究^[24]^，因此建立LCO与TME共培养模型有利于更准确地探索肿瘤的发生发展机制和创新药物的研发。Dijkstra等^[25]^研究证明了自体肿瘤类器官和外周血淋巴细胞的共培养可以使用NSCLC患者外周血中富集的肿瘤反应性T细胞，该研究为分离肿瘤反应性T细胞提供了有效的策略。Del Bufalo等^[26]^发现与成纤维细胞共培养的LCO生长得更快，表明基质成分的存在增强了肿瘤的增殖和侵袭性，该研究同时发现共培养系统中成纤维细胞与内皮细胞的结合会进一步增强肿瘤细胞的增殖和侵袭。Neal等^[27]^使用空气液体界面（air-liquid interface, ALI）类器官模型成功培养了包含有免疫细胞在内的LCO，更真实地在体外还原了TME的多样性。随着类器官培养技术的进步，也会有更多的类器官共培养模型体现原发肿瘤的免疫微环境，这些LCO与TME共培养模型在深入了解肿瘤免疫学基础和制定个性化免疫治疗策略中发挥了重要的作用。

### 3.3 微流控芯片在LCO构建的应用

传统类器官模型因稳定性差、TME成分简单、通量低等固有缺陷限制了其进一步临床转化和应用。与传统类器官培养系统相比，微流控技术可以在纳米尺度上精确控制设备的理化参数，重塑TME的理化性质，模拟TME中细胞之间复杂的相互作用。因此，在类器官微流控芯片上对患者的肿瘤细胞和免疫细胞进行共培养可以克服传统类器官功能测试的局限性^[28]^。微流控芯片可以通过调节剪切应力、间质液流动和各种自分泌或旁分泌细胞因子，从而影响类器官形态、生长速率和代谢活性。此外，类器官芯片在保留原始肿瘤遗传特征的同时，可以为LCO提供稳定的营养浓度或氧气来增加细胞活性和同质性，使得类器官相关研究能够在更接近体内的TME和受控环境中进行，为研究更复杂的生物过程与疾病建模提供创新的方法^[29]^。Poletti等^[30]^开发的微流体装置可以将类器官与从宿主分离的复杂细菌群落结合在一起，这种培养模式不仅能定期输送营养物质，通过灌注清除培养腔中脱落的死细胞，而且能构建“宿主细胞-微生物”相互作用，在延长了类器官的生存周期的同时推动基于微生物组的肿瘤治疗的研发。因此微流控芯片通过精准控制流体流动进一步改善LCO的TME，实现高通量和自动化的药物敏感性实验，为肺癌的个性化诊断和治疗提供了创新的方法^[31]^。

### 3.4 组织工程技术在LCO构建中的应用前景

除微流控芯片的应用之外，仿生支架模型培养法、水凝胶支架培养法和三维生物打印法、细胞传感器等相关工程技术在LCO的培养过程中也具有潜在的应用前景。相较于传统的基质胶，生物支架材料不仅能通过构建三维立体空间实现研究者对关键成分的精准控制，而且可以作为载体为细胞提供必要的生长因子，更精准地模拟细胞在体外的自然生长环境。Mondrinos等^[32]^使用3D模型探索了肺癌对骨骼肌的影响，并使用顺铂耐药A549细胞系在I型胶原水凝胶上建立了化学治疗耐药肺癌模型。3D打印技术作为新兴的技术体系，有望更高效地建立生物学特征高度一致且具有可重复性的LCO，取代人工方法并进一步构建更复杂的组织结构^[33]^。此外，类器官在进行药敏筛查过程中目前常用的细胞活力检测方法是腺苷三磷酸（adenosine triphosphate, ATP）生物发光技术，而细胞传感器相较于传统的ATP活性检测方式，能够实现在不破坏类器官结构的情况下，实时稳定记录细胞的生理活动状态^[34]^。但如何进一步提高LCO的稳定性和自动化程度仍然需要研究者进一步探索。

## 4 LCO在肺癌精准医学中的应用及其研究进展

### 4.1 LCO用于抗肿瘤药物的开发和应用

与传统的临床前肿瘤模型相比，LCO在研究疾病的发病机制和抗肿瘤药物的研发中具有独特的优势。Chen等^[35]^利用LCO证明活性氧（reactive oxygen species, ROS）在上皮间充质转化（epithelial-mesenchymal transition, EMT）以及细胞侵袭和迁移中发挥着关键作用，并揭示了Fangchinoline作为一种小分子药物，通过直接靶向烟酸腺嘌呤二核苷酸磷酸氧化酶4（nicotinamide adenine dinucleotide phosphate oxidase 4, NOX4），有效降低细胞质ROS水平并抑制Akt-mTOR信号通路，从而逆转NSCLC细胞的EMT，抑制细胞侵袭和迁移。该研究利用LCO进一步证明Fangchinoline能作为一种新型NOX4抑制剂，可显著抑制NSCLC的进展和转移。此外，LCO还可以用于探索肺癌的表观遗传学改变，表观遗传调节剂是有前景的抗癌靶标，但特定的抑制治疗策略尚未在体内得到证实。Sca-1（+）、CD24（+）双阳性的肺腺癌具有较高的增殖和侵袭倾向，Rowbotham等^[36]^利用LCO发现这种侵袭性表型是由H3K9甲基转移酶G9a控制的，发现H3K9甲基转移酶G9a是肺癌细胞中肿瘤增殖细胞表型的抑制因子。

### 4.2 LCO用于靶向药物的筛选

LCO保留了原发肿瘤细胞的遗传学特征，为探索肿瘤对靶向药物的敏感性和耐药性提供了一个极佳的体外预测模型^[18]^。Kim等^[37]^通过构建LCO在体外再现原发肺肿瘤的组织学结构，并在扩增过程中维持了原发肿瘤的遗传特征，该研究观察到具有不同基因突变的LCO分别对相应的药物产生反应：乳腺癌易感基因2（breast cancer susceptibility gene 2, BRCA2）突变类器官对奥拉帕尼产生药物反应、表皮生长因子受体（epidermal growth factor receptor, EGFR）突变类器官对厄洛替尼产生药物反应，EGFR突变/间质-上皮细胞转化因子（mesenchymal-epithelial transition factor, MET）扩增类器官对克唑替尼产生药物反应。Yun等^[38]^利用含有EGFR 20号外显子插入突变（EGFR exon 20 insertion mutations, Exon20ins）的Ba/F3细胞以及LCO和PDX模型，研究了Amivantamab的抗肿瘤机制，结果显示Amivantamab能够有效降低EGFR和MET介导的信号传导，并通过增加干扰素-γ（interferon γ, IFN-γ）来诱导免疫相关的抗肿瘤活性以抑制肿瘤增殖，该研究认为Amivantamab有望成为Exon20ins的有效靶向药物。Kim等^[39]^研究团队从晚期NSCLC中创建了84个LCO，并对匹配患者肿瘤标本进行了全外显子组测序分析和转录组学测序，测试它们对单一或联合靶向治疗的反应，研究结果表明LCO能够准确反映接受酪氨酸激酶抑制剂（tyrosine kinase inhibitor, TKI）治疗的NSCLC的无进展生存期和总体生存期。因此，LCO具备预测患者对靶向治疗敏感性的能力，有望提高个体化治疗的疗效。

### 4.3 LCO用于免疫药物的筛选

随着研究者对免疫检查点阻断的深入理解，以程序性死亡受体1/程序性死亡配体1（programmed cell death protein 1/programmed death ligand 1, PD-1/PD-L1）为代表的免疫治疗策略在肺癌精准医学中扮演着越来越重要的角色。现有的LCO与TME共培养技术能一定程度模拟TME，Neal等^[27]^利用ALI成功构建出具有LCO与免疫细胞共培养模型，并使用纳武利尤单抗观察LCO对免疫药物的反应效果，表明LCO可以用于免疫药物的筛选。Della等^[40]^探索了MEK和PD-L1联合抑制在NSCLC体外模型中的疗效，研究者利用LCO研究了MEK抑制剂（mitogen-activated protein kinase inhibitor, MEK-I）对NSCLC患者的PD-L1和主要组织相容性复合物I（major histocompatibility complex class I, MCH-I）蛋白表达产生的影响，确定了免疫疗法与MEK-I相结合的基本原理，显示了MEK和PD-L1抑制的协同作用。因此，利用LCO进行免疫药物的筛选有助于预测患者对不同免疫治疗策略的敏感性，并帮助研究者深入探索免疫药物的作用机制和分析药物与肿瘤细胞、TME潜在的联系。

### 4.4 LCO助力抗体药物偶联物的临床研究

在精准医学的时代，除了现有的靶向治疗、免疫治疗、抗血管治疗以及传统的化疗，抗体药物偶联物（antibody-drug conjugate, ADC）的问世引起了研究者的广泛关注。ADC由单克隆抗体、连接子和小分子细胞毒性药物（有效载荷）3部分共价偶联而成，具有单抗药物的高靶向性及细胞毒药物的高效杀伤能力等特点^[41]^。但是不同的ADC药物对不同的患者其疗效存在异质性，ADC在临床中的应用仍然受到脱靶毒性、靶向毒性等限制^[42]^。Hong等^[43]^建立了6例人表皮生长因子受体2（human epidermal growth factor receptor 2, HER2）表达水平存在差异的膀胱癌类器官，研究结果显示HER2高表达的膀胱癌类器官细胞死亡率显著高于HER2低表达类器官（48.77% vs 10.89%, P<0.01）；细胞增殖测定显示，RC48-ADC对HER2高表达的膀胱癌类器官比HER2低表达的类器官具有更强的抑制作用（11.71% vs 77.21%, P<0.01），RC48-ADC是一种新型靶向HER2的ADC药物，研究人员借助类器官证明了RC48-ADC对HER2阳性的肿瘤细胞具有良好的抗肿瘤作用。因此类器官可以作为肿瘤的“体外替身”用于ADC药物的筛选及评价。虽然LCO用于ADC药物的筛选与评价在现阶段暂未开展大规模的临床研究，但是随着ADC等新型药物在肺癌领域的应用，LCO也将在该领域给肺癌患者治疗策略的选择带来更多新的突破。

### 4.5 利用LCO探究药物的耐药机制

在晚期肺癌的治疗过程中，耐药的发生依然是治疗失败的主要原因之一。LCO在探究药物的耐药机制中具有非常重要的价值，通过建立药物敏感和药物耐药的类器官模型可以预测在临床实践中可能观察到的结局，帮助我们了解药物耐药的发生机制及其应对策略^[44]^。Yan等^[45]^研究发现双肾上腺皮质激素样激酶1（doublecortin-like kinase 1, DCLK1）通过Wnt/β-Catenin信号通路维持肿瘤细胞的干性，从而导致EGFR-TKI在肺腺癌中产生耐药，该研究表明抑制DCLK1活性可恢复对TKI耐药的肿瘤细胞和LCO的敏感性，DCLK1抑制剂与EGFR-TKI联合使用对控制肿瘤生长具有协同作用。耐药类器官可以直接从抗癌药物耐药的肿瘤患者身上获得，也可以在体外通过抗癌药物诱导类器官产生耐药。Ukai等^[46]^从手术切除的标本中成功构建了5-FU耐药性胃癌类器官，研究发现KHDRBS3的过表达导致对抗癌药物的耐药性增加，促进肿瘤的生长与转移。因此LCO有助于我们理解耐药机制和预测治疗效果，发现有意义的预后生物标志物和相关的治疗靶点，为实现更精准的个性化治疗方案提供新的思路。

## 5 LCO局限性

LCO在精准医学中具有广阔的应用前景，有望成为基础和临床肿瘤研究的理想体外模型，但目前仍然存在一些挑战：（1）LCO培养方法的差异：迄今为止研究者的类器官培养方案各不相同，例如组织消化程序、培养基组成和培养物传代条件等，因此要想将LCO在临床中成熟应用，需要研究者将这些程序标准化，并使用特定方法对培养结果进行验证；（2）LCO血管化：现阶段开发的类器官由于缺少血管结构，导致营养和氧气等难以到达类器官的核心位置，并且无法及时清除代谢废物，从而限制了LCO的生长速度和大小，甚至在培养后期引起广泛的细胞死亡，有相关研究者尝试通过整合内皮细胞或工程培养技术来解决这个问题^[47]^；（3）肿瘤的异质性：不同的肿瘤具有非常复杂的特异性TME，目前的类器官技术还不能完全准确复制患者的特异性免疫环境^[3]^；（4）正常气道上皮细胞的过度生长：大多数LCO在培养过程中存在正常气道上皮细胞过度生长，从而降低了LCO构建的成功率，这种现象可能与样本取材时的正常细胞与肿瘤细胞占比存在联系^[48]^。

## 6 展望

LCO保留了原发肿瘤的生物学特征与TME，是指导抗癌用药的强有力的工具，能避免不必要的药物试错和毒副反应，为创新药物的研发和治疗策略的选择提供了个性化的体外模型，并在预测药物反应和探究耐药机制中发挥独特的作用，有望为肺癌患者提供更精准的综合治疗策略。LCO作为精准医学领域的新兴工具，采用与创新组织工程技术相结合的方式，能够进一步在生物医学领域和个性化精准医学方面发挥独特的应用价值。
